# A pilot study of leukocyte expression patterns for drug metabolizing enzyme and transporter transcripts in autoimmune glomerulonephritis 

**DOI:** 10.5414/CP201972

**Published:** 2014-02-19

**Authors:** Melanie S. Joy, Brittney V. Roberts, Jinzhao Wang, Yichun Hu, Susan L. Hogan, Ronald J. Falk

**Affiliations:** 1 University of Colorado, Skaggs School of Pharmacy and Pharmaceutical Sciences, Aurora, CO, and; 2 University of North Carolina, Division of Nephrology and Hypertension, UNC Kidney Center, Chapel Hill, NC, USA

**Keywords:** glomerulonephritis, drug metabolizing enzymes, transporters, uridine glucuronosyltransferases (UGTs), cytochrome P450 2B9, ABCB1, ABCC2, ABCG2

## Abstract

Objective: Leukocyte mRNA expression patterns of drug metabolizing enzyme genes and transporter genes that are relevant for the disposition of cyclophosphamide and mycophenolate were studied. The relationships between expression and patient-level data and pharmacokinetics were evaluated. Methods: The study included patients with glomerulonephritis secondary to lupus nephritis (SLE, n = 36), small vessel vasculitis (SVV, n = 35), healthy controls (HC, n = 10), and disease controls (VC, n = 5; LC, n = 5). Transcript assays targeted metabolizing enzymes (*UGT1A7*,* UGT1A9*,* UGT2B7*,* CYP3A4*,* CYP2C9*, *CYP2B6*) and transporters (*ABCB1*,* ABCC2*,* ABCG2*,* SLCO1A2*). Genotyping for specific variants was conducted. Group transcript fold-changes were evaluated. Patient level data was evaluated for transcript fold-change and disease, treatment, gender, race, and genotype. Results: Significant differences were noted in expression of *UGT1A7*, *ABCB1*, and *ABCC2;* for *UGT1A7*, SVV (0.17 ± 0.42; p < 0.05) and SLE (0.03 ± 0.1; p < 0.05) groups had lower expression than HC (0.79 ± 2.02). For *ABCB1*, SLE had a lower expression (0.33 ± 0.21; p < 0.05) than HCs (1 ± 0.82). For *ABCG2*, SVV group had a lower expression (0.17 ± 0.14; p < 0.05) than HCs (1 ± 1.82). Differences in expression of *ABCC2* approached statistical significance with VC patients (2.02 ± 1.13) exhibiting higher expression than SVV patients (1.06 ± 1.11; p = 0.05). The relationships between transcript expression and patient-level data demonstrated; *ABCC2* expression was different by race (1.26 ± 1.82 Caucasian versus 1.37 ± 0.86 non-Caucasian; p = 0.049) and *CYP2B6* expression was different by treatment (2.07 ± 2.94 cyclophosphamide versus 0.45 ± 0.5 mycophenolate; p = 0.01). Conclusions: The current study showed differential expression of drug metabolizing enzyme and transporter transcripts and contributes to the literature on transcript expression of drug transporters in leukocytes. The implications of altered local metabolism and transport in leukocytes may be important in autoimmune diseases and transplant patients where treatment is targeted to leukocytes.

## Introduction 

The mRNA expression patterns of drug metabolizing enzymes and transporters in peripheral blood cells (neutrophils, lymphocytes, and monocytes) are thought to be important in patient responses to treatments for glomerulonephritis since the localized target of the pharmacological agents (e.g., mycophenolate and cyclophosphamide) are the leukocytes. The active therapeutic component of mycophenolate (mycophenolic acid) is transformed to inactive glucuronide metabolites after exposure to uridine diphosphate glucuronosyltransferases (UGTs). The pharmacologically active 4-hydroxycyclophosphamide metabolite of cyclophosphamide is first formed by phase I metabolism through cytochrome P450 enzymes and further converted to the phosphoramide mustard. Alterations in expression of drug metabolizing enzymes or transporters in leukocytes can affect the exposure of these cells to pharmacologically active components such as mycophenolic acid and 4-hydroxycyclophosphamide. 

Drug transporters would be most important to characterize, since enhanced activity and/or expression of cellular efflux genes and their respective proteins relative to uptake would reduce the intracellular concentration of therapeutic entities. Decreased activity and/or expression of efflux genes relative to uptake would increase intracellular drug concentrations. For leukocyte metabolism processes, expression of drug metabolizing enzymes may modulate the exposure of the tissue to active (4-hydroxycyclophosphamide) versus inactive (mycophenolic acid glucuronide) pharmacologic moieties. Previous studies have described the presence of *UGT* mRNA in various solid organs (liver, kidney, intestine, lung, stomach, brain, breast, prostate, heart, adrenals, bladder, ovary, uterus, and testis) within rats and humans [1, 2, 3, 4]. Metabolism processes in leukocytes, generally, would be predicted to be less contributory than systemic metabolism. The peripheral blood cells have been largely ignored for assessment of drug metabolism genes and limited studies have reported mRNA expression of selected transporters and cytochrome P450s [5, 6, 7]. These studies reported limited correlation between expression in leukocytes and intestine and liver and altered expression in subsets of leukocytes [5, 6, 7]. However, the importance of leukocyte expression and function for drugs targeting these cells for their therapeutic effects was reported by Meaden et al. [8] employing ritonavir and saquinavir. The authors reported lower MRP1 expression and higher leukocyte accumulation of ritonavir and saquinavir. They also reported higher ritonavir accumulation and lower P-glycoprotein expression. There is currently limited information regarding expression of drug transporter genes or drug metabolizing genes in patients representing specific disease models or in selected tissues (such as leukocytes) that are the targeted pharmacological site of action. 

Several exogenous and endogenous factors may be responsible for altering mRNA expression and subsequent exposure to therapeutic agents at their active site. Inducers of transport and metabolism pathways have been shown to concordantly increase activity and mRNA expression within hepatocytes [9]. mRNA expression of drug transporters has been reported to be affected by inflammatory diseases and conditions (e.g., rheumatoid arthritis, ulcerative colitis, ischemia-reperfusion injury) and direct exposure to inflammatory cytokines (e.g., TNF-α, IL-6) [10, 11, 12, 13]. Gender specific effects on *UGT* mRNA expression in tissues (liver, kidney, lung, intestine, brain, nose) have been documented in mice [14, 15]. A genotype dependent down-regulation of mRNA expression and protein function in peripheral blood mononuclear cells has also been reported in humans, whereby wild-type and heterozygotes for the *C3435T* single nucleotide polymorphism in the multidrug resistance protein gene (*ABCB1; MDR1*) exhibited less relative expression as compared to the homozygous variant genotype [16]. 

In order to incorporate clinical relevance to mRNA expression assessments, we were interested in evaluating leukocyte expression patterns of specific drug transport and metabolism genes known to be relevant in the disposition of mycophenolic acid and cyclophosphamide. Both of these compounds are clinically used in the treatment of glomerulonephritis secondary to systemic lupus erythematosus (SLE) or small vessel vasculitis (SVV). The drug transporter genes targeted in this study were *ABCB1*,* ABCC2*,* ABCG2*, and *SLCO1A2*. The drug metabolism genes targeted were *UGT1A7*,* UGT1A9*,* UGT2B7*,* CYP2C9*,* CYP2B6*, and *CYP3A4*. The purpose of conducting this pilot study was to inform about how mRNA expression data representing transporter and metabolism genes is related to patient level data and pharmacokinetic responses in autoimmune-mediated glomerulonephritis being treated with either mycophenolate or cyclophosphamide. 

## Material and methods 

### Subjects and specimens 

Patients with glomerulonephritis secondary to SLE (n = 36) and SVV (n = 35) who participated in prospective pharmacokinetic studies to evaluate mycophenolic acid [17, 18] and cyclophosphamide [19] had 15 mL of blood drawn into multiple ethylenediaminetetraacetic acid (EDTA) vacutainer tubes. The blood was obtained prior to administration of cyclophosphamide and mycophenolate. Leukocytes were isolated from blood by incubation (11 minutes) in a hypotonic red cell lysis buffer, followed by centrifugation and a wash with Hank’s balanced salt solution (HBSS). The leukocytes were subsequently lysed in RNA Stat 60 solution and stored at –70 °C for up to 2 weeks until processing. The study and consent forms were approved by the Biomedical Review Board at the University of North Carolina at Chapel Hill. 

### mRNA isolation 

The mRNA isolation procedure consisted of adding 200 µL chloroform for phase separation. The aqueous phase (containing the mRNA) was added to a solution of isopropanol and centrifuged. The pellet was then washed with 1 mL 75% ethanol, re-suspended in 100 µL nuclease free water (Promega, Madison, WI, USA), and centrifuged. Four microliters RNA secure 25X (Ambion, Austin, TX, USA) were added to each sample. The RNeasy kit and protocol (Qiagen, Valencia, CA, USA) was used for the remainder of the mRNA preparation. Briefly, after adding Buffer RLT, β-Mercaptoethanol, and 100% ethanol to the samples, the mRNA solution was applied to an RNeasy mini spin column for purification. mRNA was re-treated with RNA secure at 1X (Ambion) after the column elution. mRNA was quantified by evaluation of the absorbance at 260 nm and 280 nm using a spectrophotometer. The mRNA integrity was determined by visualization of the 28S and 18S mRNA bands using 0.5 µg mRNA on a 1% agarose gel stained with Sybr Gold (Molecular Probes, Eugene, OR, USA). mRNA was stored at –70 °C. 

### Evaluation of transcript levels 

An aliquot of each patient’s mRNA was converted to cDNA via the High Capacity cDNA Reverse Transcription kit (Applied Biosystems). A 20 µL reaction was prepared that included; 2 µL of 10x RT Buffer; 0.8 µL of 25x dNTP Mix (100 mM); 2 µL of 10x RT Random Primers; 1 µL of MultiScribe Reverse Transcriptase; 4.2 µL of Nuclease-free water and 10 µL of mRNA. The plate was placed in a thermal cycler under the profile; 25 °C for 10 minutes, 37 °C for 120 minutes, 85 °C for 5 minutes, and 4 °C for infinity. 

Pre-designed assays containing primers and probes for the assessment of transcript levels of the targeted metabolizing enzymes (*UGT1A7*,* UGT1A9*,* UGT2B7*,* CYP3A4*,* CYP2C9*, and CYP*2B6*) and transporters (*ABCB1*,* ABCC2*,* ABCG2*, and *SLCO1A2*); *UGT1A7* (Hs02517015_s1), *UGT2B7* (Hs02556232_s1), *UGT1A9* (Hs02516855_sH), *CYP3A4* (Hs00604506_M1), *CYP2C9* (Hs00426397_m1), *CYP2B6* (Hs00167937_g1), *ABCC2* (Hs00166123_m1), *ABCB1* (Hs00184500_m1), *ABCG2* (Hs01053795_m1), and *SLCO1A2* (Hs01072338_m1) were purchased from Applied Biosystems. Cytochrome C oxidase was used as the normalization (housekeeping) gene. The forward and reverse primers were designed using Primer Express software (Applied Biosystems). The forward primer (TGGCATCTGGAGGTGGTGTT) and reverse primer (GTCCAGTCCCTTTGCAGC) were purchased from Applied Biosystems. Sybr 1:400 was used as the probe in the cytochrome c oxidase assay (Molecular Probes, Leiden, Netherlands). 

Taqman^®^ PCR was performed on an Applied Biosystems PRISM 7900 HT sequence detection system (Applied Biosystems). The duplicate 10 µL reactions were performed in MicroAmp Optical 384 well plates. For the commercial assays, the reaction mixture was composed of 40 ng (4 µL) of cDNA; 0.5 µL of 20x probe and primer (Applied Biosystems), 0.5 µL nuclease-free water, and 5 µL of 2x Universal PCR Master Mix (Applied Biosystems). For the cytochrome C oxidase assay, the reaction mixture was composed of 40 ng (4 µL) of cDNA; 0.1 µL of 5 µM forward primer; 0.1 µL of 5 µM reverse primer; 0.3 µL of 1 : 400 dilution Sybr Green (Molecular Probes, Leiden Netherlands); 0.5 µL nuclease-free water, 5 µL of 2x Universal PCR Master Mix (Applied Biosystems). The thermal cycling conditions were; 50 °C for 2 minutes, 95 °C for 10 minutes, 95 °C for 15 seconds in 50 cycles, 60 °C for 1 hour. 

### Genotype assessments 

A 5 mL whole blood sample was collected into an EDTA containing vacutainer tube and genomic DNA was isolated using a Flexigene Qiagen kit (Qiagen). Genotyping was conducted for several published *UGT* single nucleotide polymorphisms (*UGT1A9*,* UGT1A7*, and *UGT2B7*) relevant for alterations in metabolism [20, 21, 22, 23, 24], and *ABCB1/MDR1* relevant for transport of mycophenolic acid [25], as previously described [26]. Genotyping was also conducted for polymorphisms in some cytochrome P450 genes (*CYP2B6*,* CYP3A4*,* CYP2C9)* relevant for alterations in cyclophosphamide metabolism [27, 28, 29]. Genotyping assessments for *CYP2B6 C1459T* (c30634242) and *CYP2B6 G516T* (c22275631) were conducted using commercially available assays (Applied Biosystems). Allelic discrimination was assessed for all Applied Biosystems products as previously described [26]. Genotypes for polymorphisms in *ABCC2*,* ABCG2* and *SLCO1A2* were not assessed. 

### Data analyses 

Stored mRNA from healthy controls; HC (n = 10), untreated SLE nephritis patients; LC (n = 5) and untreated SVV with nephritis; VC (n = 5) patients were used as study and disease controls, respectively. Ct, ΔCt, ΔΔCt, and 2^-ΔΔCt were calculated [30]. The Ct values (the fractional cycle at which the fluorescence intensity equals the threshold fluorescence; inversely related to the abundance of transcript in a sample) were computed for each sample. Subsequently ΔCt values were calculated for each sample by subtracting the Ct value for the housekeeping gene (cytochrome C oxidase) from the Ct value for the gene of interest. In order to calculate fold-change, the 2^-ΔΔCt were computed. The ΔΔCt values were calculated by subtracting the ΔCt of a selected healthy control from the ΔCt of each discrete sample. The fold-change was calculated by dividing the individual 2^-ΔΔCt values by the average of the 2^-ΔΔCt values for healthy control samples. 

Transcript fold-change in each of the five groups (SVV, VC, SLE, LC, HC) were computed and recorded as medians, and mean ± standard deviation. Significant differences between the median fold-change values among patient groups were determined using Kruskal Wallis nonparametric ANOVA. A post-ANOVA Dunn’s Multiple Comparison’s test was used to determine differences in median transcript expression. Patient level data that was evaluated for relationships with transcript fold-change were: disease (SVV vs. SLE), treatment (cyclophosphamide vs. mycophenolate), gender, race (Caucasian vs. non-Caucasian), and genotype (*UGT1A7*,* UGT1A9*,* UGT2B7*,* CYP2C9*,* CYP3A4*,* CYP2B6*, and *ABCB1*). The expression values were converted to the log 10 to enable a normal distribution so that linear regression could be used to evaluate these former relationships. Spearman correlation analysis was used to evaluate relationships between fold-change expression values by disease, genotypes, treatments, gender, and race. Spearman correlation analyses were also conducted to evaluate for relationships between continuous mycophenolic acid and cyclophosphamide pharmacokinetic variables [17, 18, 19]; area under the plasma concentration time curve (AUC), trough plasma concentration (Ctr), apparent oral clearance (mycophenolic acid), systemic clearance (cyclophosphamide), renal clearance, and transcript expression. Wilcoxon two-sample tests were used to assess for relationships of *SLCO1A2* transcript expression between gender, race, disease, and treatment. p-values of < 0.05 were considered statistically significant. Statistical analyses were performed using InStat v3.0 (GraphPad, San Diego, CA, USA) and SAS Statistical Software, Version 9.1 (SAS Institute, Inc., Cary, NC, USA). 

## Results 

The description of SLE and SVV study subjects who donated blood for gene transcript analyses are provided in Table 1. This information was not available (demographics) or did not apply (treatment) to the three control groups. The transcripts of transporter genes (*ABCB1*,* ABCG2*, and *ABCC2)* were expressed in the leukocytes of 92 – 98% of subjects. By contrast, the transcript of *SLCO1A2* was expressed in only 50% of subjects. Regarding the drug metabolizing enzyme genes, the transcripts of *UGT1A9*, *UGT1A7*, and *UGT2B7* were expressed in ~ 50% of subjects, while the *CYP2B6* transcript was expressed in 94% of subjects. The *CYP3A4* and *CYP2C9* genes were not appreciably expressed in the leukocytes of the evaluated subjects. Fold-change values for each gene in each patient group (SVV, VC, SLE, LC, HC) are recorded as mean ± SD in Table 2. Significant differences were noted in expression of *UGT1A7*, *ABCB1*, *ABCG2*, and *ABCC2* across the evaluated patient populations. Regarding *UGT1A7*, the SVV (0.17 ± 0.42; p < 0.05) and SLE (0.03 ± 0.1; p < 0.05) groups had statistically lower expression values than the HC subjects (0.79 ± 2.02). For *ABCB1*, the SLE group had significantly lower expression values (0.33 ± 0.21; p < 0.05) than the HC group (1 ± 0.82). For the *ABCG2* gene, the SVV group had lower mean expression values (0.17 ± 0.14; p < 0.05) than the HC subjects (1 ± 1.82). Differences in expression of *ABCC2* approached statistical significance, with the VC patients (2.02 ± 1.13) exhibiting higher expression than the SVV patients (1.06 ± 1.11; p = 0.05). 

Genotype frequencies for the *UGT1A7*,* UGT2B7*,* ABCB1*, and *CYP2B6* single nucleotide polymorphisms evaluated in the 67 treated SLE and SVV patients are shown in Table 3. Genotype frequencies for all evaluated polymorphisms were in Hardy-Weinberg equilibrium. Genotype analyses are not reported for the *UGT1A9* polymorphisms that were planned due to their extremely low frequency in this glomerulonephritis population. 

Several important findings (p < 0.05) resulted from the evaluation of the relationships between transcript expression and patient-level data (Table 4). However, none of the relationships resulted in R^2^ values of greater than 0.10 secondary to the dichotomous nature of the patient-level data. Among the SVV and SLE groups receiving treatment with either mycophenolate or cyclophosphamide, *ABCC2* expression was different by race (1.26 ± 1.82 Caucasian vs. 1.37 ± 0.86 non-Caucasian; p = 0.049); *CYP2B6* expression was different by treatment (2.07 ± 2.94 cyclophosphamide vs. 0.45 ± 0.5 mycophenolate; p = 0.01). Results of borderline significance (0.05 < p < 0.10) were *ABCB1* expression by *ABCB1 C3435T* genotype (0.43 ± 0.55 wildtype vs. 0.63 ± 0.88 variants; p = 0.076), *ABCC2* expression by disease type (1.20 ± 1.5 SVV vs. 1.43 ± 1.29 SLE; p = 0.078), and *ABCG2* expression within SLE patients by gender (0.34 ± 0.34 female versus 0.11 ± 0.07 male; p = 0.074). Assessment of relationships between *UGT* or *SLCO1A2* expression and patient-level variables were not attempted secondary to the higher percentage of subjects with absent transcript in leukocytes. Additionally, too few subjects exhibited the evaluated single nucleotide polymorphisms in the *UGT1A9* gene to enable evaluation with transcript expression. 

**Figure 1:**
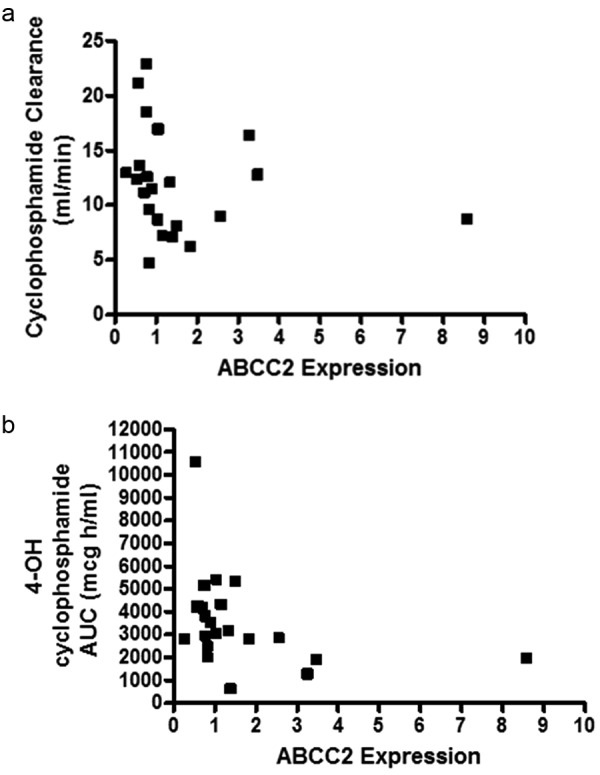
a: Correlation between leukocyte ABCC2 mRNA expression and cyclophosphamide clearance. A significant negative correlation was noted between ABCC2 mRNA expression and cyclophosphamide clearance ; r = –0.4263, 95% C.I. –0.7252 to 0.007672, p = 0.0479. b: Correlation between leukocyte ABCC2 mRNA expression and 4-OH cyclophosphamide clearance. A significant negative correlation was noted between ABCC2 mRNA expression and 4-OH cyclophosphamide clearance ; r = –0.4850, 95% C.I. –0.7585 to –0.06640, p = 0.0221.

Assessments of relationships between transcript expression and pharmacokinetic parameters for mycophenolic acid and cyclophosphamide were evaluated to ascertain whether overall dispositional effects were demonstrated. Significant negative correlations were noted between *ABCC2* expression and cyclophosphamide clearance (r -0.426; p = 0.048), and 4-hydroxycyclophosphamide AUC (r –0.4850; p = 0.0221) (Figure 1). No significant correlations were noted for mycophenolate pharmacokinetic parameters and mRNA expression data. 

## Discussion 

The current study is the first to describe expression of drug metabolizing enzyme and drug transporter transcripts in the leukocytes of patients with kidney disease secondary to immune-mediated glomerulonephritis. This research is relevant as therapies for the treatment of glomerulonephritis are primarily directed toward the peripheral blood leukocyte population. This study selectively assessed only those genes thought to be involved in the transport and metabolism of mycophenolate and cyclophosphamide, the two primary treatments in glomerulonephritis. Our studies demonstrated reasonable leukocyte expression of transporter transcripts (*ABCC2*,* ABCB1*,* ABCG2;* 90% of patients and *SLCO1A2;* 50% of patients) in the blood of glomerulonephritis patients. Reasonable expression of leukocyte *UGT* transcripts (*UGT1A9*, *UGT1A7*, and *UGT2B7;* 50% of patients), and *CYP2B6* (> 90% of patients) were noted as well. *CYP3A4* and *CYP2C9* expression was virtually absent. 

Since transporters on leukocytes have been reported to contribute to the intracellular concentrations of drugs, we were interested in evaluating the expression of drug transporters on the leukocytes of patients with glomerulonephritis [31, 32, 33, 34]. Differences in mean drug transporter transcript expression among the subject groups were found in the present study. Healthy controls had a higher *ABCB1* expression than SLE patients, and a higher *ABCG2* expression than SVV patients. These findings could contribute to higher intra-leukocyte concentrations of substrates transported by P-glycoprotein and breast cancer resistance associated protein in patients with SLE and SVV, respectively. We plan to assess intracellular concentrations of drugs in future studies that employ patients with glomerulonephritis. Albermann et al. [5], previously reported the relative order of gene expression for ABC-transporters in peripheral blood mononuclear cells as *ABCC1*>*ABCG2*>*ABCB1*>*ABCC2*. The relative order of magnitude in transcript expression for glomerulonephritis patients was *ABCC2*>*SLCO1A2*>*ABCB1* = *ABCG2* for SLE and *SLCO1A2*>*ABCC2*>*ABCB1*>*ABCG2* for SVV. This data demonstrates that the MRP2 and OATP transporters (most pertinent to overall disposition of mycophenolic acid) have the highest expressed transcripts within the leukocytes of SLE and SVV patients. Mycophenolic acid is a substrate of P-glycoprotein [25, 35, 36], and BCRP [37], the glucuronide metabolite is a substrate for MRP2 [38], and glucocorticoids are known substrates for P-glycoprotein [39, 40]. In vitro studies that assess the role of transporters of mycophenolic acid and its metabolites and leukocyte disposition will be assessed in our future studies. 

Our evaluations regarding drug metabolism genes demonstrated that healthy controls had a higher expression of *UGT1A7* relative to SVV and SLE patients. Since mycophenolic acid is a substrate for UGT1A7 [41] and SLE and SVV patients have reduced *UGT1A7* transcript expression relative to healthy controls, the glomerulonephritis patients would be predicted to have a lower metabolism of mycophenolic acid through UGT1A7 within the leukocytes. The affinity of mycophenolic acid for UGT1A7 is greater than the affinity for UGT1A9 [41], another common metabolizing enzyme, however, the overall relative contribution of UGT1A7 to mycophenolic acid metabolism in various tissues, including leukocytes has not been reported. 

In order to provide clinical relevance to our work, we were interested in exploring the effects of patient-level factors on transcript expression in the SLE and SVV patients. These factors (treatment, gender, race, and genotype) were included as existing publications supported their evaluations [9, 14, 16, 42, 43, 44]. Treatment-related differences in expression were assessed in mycophenolate- vs. cyclophosphamide-treated patients. Our results showed that cyclophosphamide-treated glomerulonephritis patients had a 4-fold higher expression of *CYP2B6* (2.07 ± 2.94 vs. 0.45 ± 0.5; p = 0.01) than mycophenolate-treated patients. While it is tempting to attribute this finding to induction of gene transcription by cyclophosphamide, this scenario is unlikely for several reasons including the fact that previous doses had been administered at least 30 days prior, doses were lower (0.8 ± 0.2 g/m^2^) than reported for enzyme induction [45], and blood was obtained prior to and not after the next planned dose. For SLE patients, higher expression of *ABCC2* and *ABCG2* would be predicted to reduce intracellular exposure to mycophenolic acid as compared to SVV patients. While we did not prospectively measure this directly, our previous pharmacokinetic publications [17, 18], do support higher systemic (extracellular) exposures in SLE vs. SVV patients. Exposures to other concurrent treatments, however, can influence expression of drug metabolizing enzyme transcripts [9, 44, 46]. Glucocorticoids were prescribed in 36% of our mycophenolate-treated and 86% of our cyclophosphamide-treated patients. Based on previous data from glucocorticoid exposures [9], induction of *ABCC2* and *SLCO1A2* can occur. The finding of high expression of both the *ABCC2* and *SLCO1A2* transcripts in our SLE and SVV patients suggests an influence from glucocorticoid exposure. 

Since recent publications have reported gender divergent effects on *UGT* transcript and tissue expression in mice [14, 15] and reduced activity of UGTs females [47], we wanted to evaluate the gender-stratified expression of our evaluated genes in the glomerulonephritis population. While none of these assessments reached statistical significance, a trend was noted in female patients having 3-fold higher expression of the *ABCG2* transcript than males. This finding is interesting as females comprise the majority of SLE patients, and higher expression of *ABCG2* was found in the SLE patients; implying that a disease-gender interaction may be confounding. Regarding our assessments of the effects of race, the expression of *ABCC2* in leukocytes was found to be lower in Caucasian than non-Caucasian SLE and SVV patients. The non-Caucasian group comprised the majority of SLE patients and these patients were disproportionately African-American. African-American SLE patients are known to have worse treatment related outcomes [48]. We plan to explore the relationships between intracellular concentrations of drug treatments, expression of transporters, and treatment outcomes in future studies. In the current study, we found higher *ABCB1* leukocyte expression in patients who exhibited the C/T and T/T genotypes as compared to the wildtype (C/C) genotype, a finding consistent with the literature [16, 49]. The role of genotype on transcript expression of *ABCB1* was recently reported in a study that isolated peripheral blood cells from healthy subjects and incubated them with lipopolysaccharide (LPS) [16]. However, the published data concerning P-glycoprotein activity in patients by genotype for the *ABCB1 C3435T* polymorphism are conflicting [49]. 

Since expression of transporters on leukocytes has been reported to alter intracellular pharmacokinetics, we wanted to evaluate the correlation between mycophenolic acid and cyclophosphamide clearance and/or exposure parameters and transcript expression. Since we did not evaluate intracellular concentrations of drugs, systemic clearance and/or exposure data was used. Significant negative correlations were found between *ABCC2* expression with cyclophosphamide clearance and with 4-hydroxycyclophosphamide AUC. Based on our pilot study, this could imply that enhanced exposure to 4-hydroxycyclophosphamide would be predicted when low *ABCC2* transcript expression is present. Previously published data has suggested that MRP2, along with MRP4 and BCRP2 contribute to the disposition of 4-hydroxycyclophosphamide [50], providing some physiological relevance to our pilot study findings. The protein of *ABCC2*, e.g., MRP2 is localized to the apical (bile cannilicular) membrane of liver and serves to efflux organic anions from hepatocytes. Decreased MRP2 protein in liver would result in reduced loss of 4-hydroxycyclophosphamide from the hepatocytes and enhanced opportunity for efflux through MRP4 at the hepatocyte basolateral membrane, with increased AUC. 

## Conclusions 

The current study showed differential expression patterns of drug metabolizing enzyme and transporter transcripts in patients with active glomerulonephritis as compared to healthy subjects and disease control subjects without active glomerulonephritis. Treatment and patient-specific variables were associated with significant differences in expression of drug metabolism and transport genes. This study adds to the sparse literature describing the transcript expression of drug transporters in leukocytes and focuses on a disease in which patients receive therapies targeted to the leukocytes. This basic knowledge is required as transcript and ultimately protein expression of drug metabolizing enzymes and transporters can modulate the exposure to active pharmacologic moieties in the blood and tissues. The data from this pilot study will guide future investigations into mechanisms for altered treatment responses. It will be necessary to validate the current study’s findings in a larger cohort of patients. Large prospectively designed studies with serial expression profiles will be necessary to validate cause and affect relationships. The implications of altered local metabolism and transport in leukocytes may be important in the treatments of other autoimmune diseases and transplantation. 

## Funding 

This research was funded by the National Institutes of Health 5K23DK64888, General Clinical Research Centers program of the Division of Research Resources, National Institutes of Health RR00046, Clinical and Translational Science Award U54RR024383, and American College of Clinical Pharmacy Research Institute’s Frontier’s Award. 

## Conflict of interest 

No conflicts of interest by any of the authors. 

**Table 1:** Demographics of glomerulonephritis patients. Data presented as n (percentage).

	Small vessel vasculitis (n = 35)	Systemic lupus erythematosus (n = 36)
Race (%)		
Caucasian	25 (71%)	8 (22%)
Non-Caucasian	10 (29%)	28 (78%)
Gender (%female)	20 (57%)	28 (78%)
Treatment (%)		
Cyclophosphamide	7 (20%)	15 (42%)
Mycophenolic acid	28 (80%)	21 (58%)

**Table 2:** Fold-change transcript values in the evaluated groups (mean ± SD).

	SVV (n = 35)	SVV-control (n = 5)	SLE (n = 36)	SLE-control (n = 5)	HC (n = 10)
*UGT1A9*	0.98 ± 2.24	NA	0.62 ± 1.27	0.34 ± 0.27	0.94 ± 1.73
*UGT2B7*	2.46 ± 6.38	0.52 ± 0	2.13 ± 4.87	1.35 ± 1.78	1.00 ± 1.64
*UGT1A7*	0.17 ± 0.42^a^	0.27 ± 0	0.03 ± 0.1^b^	0.22 ± 0.21	0.79 ± 2.02
*CYP2B6*	0.50 ± 0.57	0.15 ± 0.12	1.49 ± 2.55	0.50 ± 0.62	1.00 ± 0.99
*ABCB1*	0.65 ± 0.96	0.54 ± 0.6	0.33 ± 0.21^c^	0.45 ± 0.31	1.00 ± 0.82
*ABCC2*	1.06 ± 1.11^d^	2.02 ± 1.13	1.35 ± 1.21	1.60 ± 1.08	1.00 ± 0.41
*ABCG2*	0.17 ± 0.14 ^e^	0.01 ± 0	0.31 ± 0.33	0.10 ± 0.07	1.00 ± 1.82
*SLCO1A2*	1.45 ± 3.68	NA	0.47 ± 0.75	0.01 ± 0	0.84 ± 0.99

^a^SVV < HC; p < 0.05; ^b^SLE < HC; p < 0.05; ^c^SLE < HC; p < 0.05; ^d^SVV < SVV-control; p = 0.05; ^e^SVV < HC; p < 0.05. *ABCB1*: multidrug resistance protein; *ABCC2*: multidrug resistance-associated protein; *ABCG2*: breast cancer resistance protein; ANCA: antineutrophil cytoplasmic antibody; *CYP*: cytochrome P450; HC: healthy control; NA = not applicable; *SLCO1A2*: organic anion transporting polypeptide; SLE: systemic lupus erythematosus; SVV = small vessel vasculitis; *UGT* = uridine diphosphate glucuronosyltransferase.

**Table 3:** Genotype frequency distributions (frequency (n)).

		SLE and SVV patients
*UGT1A7*		
T622C	T/T	0.53 (35)
	T/C	0.42 (28)
	C/C	0.05 (3)
*UGT2B7*		
C802T	C/C	0.39 (26)
	C/T	0.42 (28)
	T/T	0.19 (13)
*CYP2B6*		
C1459T	C/C	0.82 (55)
	C/T	0.15 (10)
	T/T	0.03 (2)
G516T	G/G	0.49 (33)
	G/T	0.43 (29)
	T/T	0.08 (5)
*ABCB1*		
C3435T	C/C	0.34 (23)
	C/T	0.55 (37)
	T/T	0.11 (7)
C1236T	C/C	0.43 (29)
	C/T	0.49 (33)
	T/T	0.08 (5)

*ABCB1* = multidrug resistance protein; *CYP* = cytochrome P450; *UGT* = uridine-glucuronosyltransferase.

**Table 4:** Relationships between transcript expression and patient-level data in subjects with systemic lupus erythematosus and small vessel vasculitis.

Transcript variable	Patient-level variable	Parameter estimate	p-value
*ABCB1*	Gender	0.07	0.542
	Race	0.061	0.558
	Treatment	–0.049	0.66
	Disease	0.152	0.144
	*ABCB1 C3435T* genotype	–0.194	0.078
	*ABCB1 C1236T* genotype	–0.092	0.385
*ABCC2*	Gender	0.113	0.203
	Race	–0.157	*0.049*
	Treatment	0.113	0.184
	Disease	–0.141	0.078
*ABCG2*	Gender	0.224	0.093
	Race	–0.07	0.562
	Treatment	0.058	0.657
	Disease	0.04	0.831
*CYP2B6*	Gender	0.14	0.531
	Race	–0.196	0.33
	Treatment	0.537	*0.01*
	Disease	–0.142	0.483
	*CYP2B6 A785G* genotype	0.049	0.906
	*CYP2B6 C1459T* genotype	–0.166	0.533
	*CYP2B6 G516T* genotype	0.083	0.68

Transcript expression values were log 10 transformed for analyses. p-values < 0.05 are *italicized.*
